# Obesity-related asthma and its relationship with microbiota

**DOI:** 10.3389/fcimb.2023.1303899

**Published:** 2024-01-15

**Authors:** Jinli Huang, Xuehua Zhou, Bo Dong, Hong Tan, Qiuhong Li, Juan Zhang, Hui Su, Xin Sun

**Affiliations:** ^1^ Department of Pediatrics, Xijing Hospital, the Fourth Military Medical University, Xi’an, China; ^2^ Department of Geriatrics, Xijing Hospital, the Fourth Military Medical University, Xi’an, China

**Keywords:** obesity-related asthma, microbiota, mechanism, immunity, treatment

## Abstract

Obesity and asthma are global public health problems. Obesity-related asthma is a special phenotype of asthma with a complex pathogenesis. Its occurrence and development are related to mechanical compression, inflammatory response, metabolic regulation, gene regulation, and vitamin D deficiency. Different treatment strategies used in the process of weight loss have a beneficial impact on asthma. Alterations in gut and airway microbial community structure and their metabolites may also contribute to obesity-related asthma. The role of the Th17/Treg balance in the gut microbiota regulating the immune responses and host metabolism is important. Therapeutic measures associated with the gut microbiota variety may contribute to improving chronic inflammation associated with obesity by regulating the Th17/Treg balance. An early reduction in microbial diversity can predict the development of asthma and lead to allergy through an imbalance of Th2/Th1 responses. Short-chain fatty acids (SCFAs) regulate the differentiation and activation of regulatory T cells, thereby regulating immune homeostasis in the lung to suppress allergic inflammation and weight gain. Therefore, clarifying the microbial mechanism of obesity-related asthma has important guiding significance for clinical treatment. In this review, we used the following terms: “asthma and obesity” and “obesity-related asthma”, combining “phenotype”, “airway inflammation” and “lung function”, and reviewed the characteristics and pathogenesis of obesity-related asthma, the relationship between the gut and airway microbiota and obesity-related asthma, and the current treatment measures for the disease.

## 1 Introduction

Rapid urbanization and globalization have contributed to the obesity epidemic (Kaur et al., 2015; Wang et al., 2020). Overweight and obesity are defined by the World Health Organization (WHO) as abnormal and excessive fat accumulation that may compromise health. WHO estimates that no less than 1.4 billion people over the age of 20 are overweight (Barros et al., 2017). Obesity is associated with an increased risk of diabetes, osteoarthritis, cardiovascular disease, and various malignancies (Ekström et al., 2017; Sommer and Twig, 2018). Asthma is a chronic respiratory disease characterized by airway inflammation and hyperresponsiveness. In recent decades, the prevalence has continued to rise as Western dietary patterns have become more common, and affecting more than 300 million individuals worldwide (Côté et al., 2020; Kopsaftis et al., 2021).

Obesity is a major risk factor for asthma in children and adults, and the mechanistic basis of this relationship remains unclear (Lundgren et al., 2021). Asthma is a heterogeneous disease with different basic illness processes and different asthma phenotypes. Evidence suggests that obesity and asthma are related closely and affect each other’s natural processes significantly (Miethe et al., 2020). Numerous studies have shown that obesity is closely associated with increased risks of asthma in adults and children (Kaur et al., 2015; Piche et al., 2020). The strongest predictor of childhood asthma development is rapid weight gain during the first two years of life (Rzehak et al., 2013). Asthma-related exercise intolerance and poor quality of life may also contribute to more obesity. Therefore, studying the interaction of obesity and asthma becomes crucial.

The Global Initiative for Asthma (GINA) pointed out that asthma has an important special phenotype: obesity-related asthma. Patients with this phenotype have obvious respiratory symptoms, but airway eosinophilic inflammation is not obvious (Hering, 2017). This phenotype is dominated by neutrophil infiltration, is not sensitive to inhaled corticosteroids, and is prone to developing refractory asthma. Obesity-related asthma increases the risk of hospitalization for patients with severe disease and has poor efficacy with standard treatment regimens (Hassan et al., 2020). Obesity-related asthma is a syndrome that includes different disease phenotypes. At present, it is found that obesity-related asthma mainly includes two simple phenotypes: one is caused by obesity and has a delayed onset, which is late-onset asthma; the other is early-onset. Late-onset asthma has a low inflammatory response, and airway hyperresponsiveness gradually improves with weight loss; the other one has an earlier onset and a higher allergic inflammatory response, and the condition can be aggravated by obesity (Sukhan, 2018). Microbes are an important part of maintaining homeostasis in the body, and obesity-related alterations in the gut and/or airway microbial diversity enlarge the risk of asthma in obese individuals (Dupont et al., 2020).

The aim of this article is to summarize the data that supports the relationship between obesity and asthma, to describe the clinical features of patients with obesity-related asthma, to explore its pathogenesis, to analyze its relationship with the microbiota, and to clarify different treatment options that may improve patient outcomes.

## 2 Methods

In order to discuss the pathogenesis of obesity-related asthma and its relationship with the microbiota, we undertook a systematic literature search that included clinical research and experimental studies. Searches were conducted using PubMed, Google Scholar, and Medline with the following key terms: obesity, asthma OR obese asthmatic mice AND microbiota, inflammation OR obesity-related asthma AND mechanism, airway hyperresponsiveness, a treatment OR immunity AND medication. Studies from all years were included, specifically those published within the last 10 years. Some review articles and their reference lists were also searched to identify related articles.

## 3 Obesity is a risk factor for asthma

The prevalence and incidence of both obesity and asthma are on the rise, and having both in the same person is not a simple coexistence of two common conditions. In the United States, asthma affects nearly 6.5 million kids (approximately 9% prevalence) (The Lancet, 2018), and 15% of kids are overweight and another 17% are obese (Dabas and Seth, 2018). Now, obesity is recognized as a main risk factor for asthma; some longitudinal epidemiological studies have shown that obesity usually precedes asthma attacks. Obesity is also associated with increased asthma severity, and obesity-induced increased asthma risk may begin *in utero*. A meta-analysis indicated maternal obesity and overweight during pregnancy were found to connect with increased asthma risk in offspring (15- 30%); other reports have similar findings (Liu et al., 2020); and mechanisms involved in this risk may include inflammation or other changes during pregnancy and early postpartum (Bar-Noy et al., 2021).

Another meta-analysis of prospective studies of more than 300,000 adults showed that the odds of developing asthma were 1.9% in the obese group and 1.5% in the overweight group compared with the lean group. Actually, in the United States, 250,000 asthma cases are associated with obesity each year (Miethe et al., 2020), and the U.S. asthma demographics found an asthma prevalence of 7.1% in thin adults and 11.1% in obese adults. This relationship was more pronounced in women, with asthma prevalence rates of 7.9 percent and 14.6 percent in thin and obese women, respectively (Akinbami and Fryar, 2016). Among adults, the prevalence of asthma is 2 to 3 percent higher in obese individuals than in overweight or normal-weight individuals (Akinbami et al., 2018). Up to 60 percent of patients with severe asthma are obese (Schatz et al., 2014). In obesity-related asthma patients, weight loss can significantly reduce asthma symptoms and reduce airway hyperreactivity (AHR) (Oppenheimer et al., 2020). Studies found that for obesity-related asthma patients, it is difficult to achieve asthma control (Jiang et al., 2020).

Among adolescents who participated in the National Health and Nutrition Examination Survey (NHANES), obesity was connected with asthma only in participants with low or normal exhaled nitric oxide and was associated with increased asthma severity in those who already had asthma and high FeNO. Obesity complicates classic T helper 2 cells (Th2) inflammation; e.g., in obesity, eosinophilic airway inflammation is altered and eosinophil transport into the airway lumen is altered, and obesity biases CD4^+^T cells toward T helper 1 cell (Th1) polarization (Phull et al., 2017). Notably, eosinophils and alternately activated macrophages play important roles in healthy adipose tissue homeostasis. Infiltration of pro-inflammatory M1 macrophages and reduction of eosinophils are associated with obesity and insulin resistance, whose relationship to airway disease is unclear. Innate lymphoid cells (ILCs) play a significant role in the homeostasis of adipose tissue; therefore, changes in the function of ILCs in adipose tissue may lead to obesity and asthma. Meanwhile, activation of macrophages through ILCs and other pathways may establish an important link between adipose and asthma exacerbation outcomes (Jiang et al., 2019).

Changes in lung innate immune function may promote obesity-related asthma. For instance, obesity-related asthma patients have lower levels of surfactant-associated protein A (SPA) in the bronchoalveolar fluid compared with lean people, which helps regulate responses to infections and other injuries. Eosinophils are elevated in the submucosa in a subgroup of obese asthmatics. Surfactant protein-A (SP-A) modulates host responses to infectious and environmental insults. Lugogo et al. used SP-A^−/−^ mice to challenge allergen models, and exogenous SPA therapy was given after the last challenge. They found allergen challenged SP-A^−/−^ mice that received SP-A therapy had significantly less tissue eosinophilia compared to mice receiving vehicle (Lugogo et al., 2018). According to a cross-trait genome-wide association study (GWAS), which was performed using 457,822 subjects of European ancestry from the UK Biobank. They found a substantial positive genetic correlation between BMI and later-onset asthma defined by asthma age of onset at 16 years of age or older (*Rg* =0.25, *P*=9.56×10^−22^). Cross-trait meta-analysis identified 34 shared loci among 3 obesity-related traits and 2 asthma subtypes. GWAS functional analyses identified potential causal relationships between the shared loci and genotype-tissue expression (GTEx) tissue expression quantitative trait loci (eQTLs), shared immune- and cell differentiation-related pathways between obesity and asthma. The study reinforces the hypothesis that obesity causally increases the risk of asthma (Zhu et al., 2020).

## 4 Interaction between obesity and asthma

Among recent longitudinal studies found that obesity precedes asthma, a large study using data from the Taiwan Children Health Study reported that obesity showed the highest risk of incident asthma (hazard ratio [HR] = 1·28; 95% CI: 1·05, 1·56). The study also suggests that obesity and obesity-related risk factors (sleep breathing disorders and sleep quality) are associated with asthma (Chen et al., 2022). Another study found that fat gain before age 6 was the most sensitive in predicting childhood asthma, while prepuberty was the most predictive of youthful asthma (Chen et al., 2021). A recent meta-analysis showed that childhood obesity increased the odds of asthma by about 50% (Malden et al., 2021). Another meta-analysis reported that maternal obesity and weight gain during pregnancy increased the risk of asthma in infants by 15-30% (Castro-Rodriguez et al., 2020). A high BMI at age 8 was found to be associated with an increased risk of later asthma with allergic rhinitis, while being overweight/obese at age 20 was associated with a higher risk of asthma onset without allergic rhinitis (Nwaru et al., 2020). There is increasing evidence that asthma may also contribute to obesity, possible causes are reduced physical activity and frequent use of systemic corticosteroid hormones. A multicohort study reported that children with asthma had a 23% higher risk of obesity compared to non-asthmatic children (Stratakis et al., 2022). This means that better asthma control may reduce the risk of obesity. Studies have found that children with poorly controlled asthma may have increased fat mass and decreased muscle mass, independent of BMI (Abdo et al., 2021). This highlights the complexity of the relationship between obesity and asthma.

Research reported that obese girls with asthma have higher circulating levels of sCD163 (a marker of macrophage activation) are associated with lower lung function and quality of life in asthma patients (Periyalil et al., 2015). Innate lymphoid cells (ILCs) also play a role in obesity-related asthma (Everaere et al., 2018). IL17A-producing ILC3 cells are increased in the lungs of obese mice with AHR (Kim et al., 2014). Higher levels of ILC3 cells were found in the peripheral circulation of obese children with asthma but were not associated with disease severity (Wu et al., 2018). The study found leptin enhanced ILC2 survival and proliferation and enhanced symptoms of allergic asthma, whereas reduced ILC2 suppresses AHR in obese mice compared with lean mice (Zheng et al., 2016). The study showed that obesity causes Th1 inflammation and that Th1 inflammation is more intense in obese children with asthma. It also found that obesity affects asthma in both individuals with and without allergies (Nyambuya et al., 2020). Obesity has also been associated with eosinophil activation and chemotaxis in asthma (Grotta et al., 2013). Submucosal eosinophilia in obese adults with severe asthma has also been associated with obesity (Desai et al., 2013). Studies in mice have found that obesity leads to an increase in the proportion and activation of airway macrophages (Tashiro et al., 2017) and a decrease in AHR when obesity symptoms are reduced (Kim et al., 2015).

## 5 Clinical manifestations of obesity-related asthma

Common symptoms of obesity and asthma include pain, fatigue, depression, and anxiety. These symptoms lead to social isolation, decreased physical activity, and reduced quality of life, which may also lead to increased morbidity and mortality in the near future. Asthma in obese patients is more severe, more symptomatic, less controlled, poorer responsive to treatment, and those patients has a lower quality of life (Jiang et al., 2019). The prevalence of obesity in adults with severe asthma is higher than that in the general population, and the 1-year risk of asthma visits in overweight patients is 1.36, 1.50, and 3.70 (Porsbjerg and Menzies-Gow, 2017; Lugogo et al., 2018). Obese adults are 4 to 6 times more likely to be hospitalized than thin adults with asthma. Obesity is associated with longer hospital stays and a higher risk of mechanical ventilation in children and adults with asthma (Okubo et al., 2016; Barros et al., 2017). Obese patients may have other comorbidities, such as gastroesophageal reflux disease, hypertension, obstructive sleep apnea, insulin resistance, and osteoarthritis (OA) (Lugogo et al., 2018).

Gastroesophageal reflux disease, which is quite common in severe asthma and causes bronchoconstriction, represents a major risk factor for asthma exacerbations (Luthe et al., 2018). Likewise, obstructive sleep apnea (OSA) is more prevalent in severe asthma. Asthma with OSA has a higher frequency of severe exacerbations than those without OSA, is associated with systemic inflammation, and may exacerbate macrophage infiltration in obese individuals (Lugogo et al., 2018; Maio et al., 2018). As the degree of insulin resistance increases, the relationship between asthma and obesity becomes stronger, although the clear mechanism of how insulin resistance acts on asthma in obese individuals has not been determined.

## 6 Mechanisms of obesity-related asthma

### 6.1 Mechanism of mechanical effect

Lung function limitation and increased airway hyperresponsiveness have been reported as possible links between obesity and asthma in obese patients (Jung et al., 2013). People with asthma had lower lung function, higher rates of atopic disease, and a higher BMI. Obesity can affect respiratory dynamics and directly affect respiratory function. Subcutaneous fat compressing the thoracic cavity and visceral fat occupying the thoracic cavity cause all lung volumes to decrease. The reduction of expiratory reserve volume (ERV) and functional residual capacity (FRC) is proportional to the degree of obesity (Bokov and Delclaux, 2019).

### 6.2 Inflammation mechanism

Obesity was associated with increased pro-inflammatory signaling, which was enhanced in the presence of asthma. Studies have found that obesity-related asthma can increase systemic inflammation. Hepatic acute-phase protein C-reactive protein, fibrinogen, and serum amyloid A were significantly elevated in the serum of obese asthmatic patients compared with healthy volunteers. Ontologies related to inflammation and innate immune responses were significantly enriched in obese asthmatics, suggesting an additive effect between asthma and obesity.

In addition, airway inflammation is affected by obesity. Bronchoalveolar lavage levels of intercellular adhesion molecule 1 and vascular cell adhesion molecule-1 and IL-5 increased, and the total number of inflammatory cells (including eosinophils, neutral granulocytes, and lymphocytes) was significantly elevated in obese asthmatic patients (Michalovich et al., 2019).

Obesity is manifested by low-grade chronic inflammation, which may affect lung function and exacerbate asthma. Macrophages in adipose tissue play a significant role in the pathogenesis of metabolic inflammation, with increased infiltration of macrophages in visceral adipose tissue in obese asthmatics (Sideleva et al., 2012). Studies have shown that airway macrophage phagocytosis is 40% lower in obese asthmatics than in non-obese asthmatics (Fernandez-Boyanapalli et al., 2013). Furthermore, circulating regulatory T cells (Tregs) are significantly lower in obese children with asthma compared to healthy controls (Donma et al., 2015). Systemic inflammation in obese asthma is similar to but greater than inflammation in obesity alone. T helper cell type 1 (Th1) cells are recruited and activated as a result of the local and systemic inflammatory responses mediated by adipose tissue macrophages in obesity (Fantuzzi, 2005).. In contrast, typical childhood asthma is associated with a Th2 phenotype. IL-6 has been implicated in both asthma and obesity. In the Severe Asthma Research Program-3 (SARP3) adult cohort, IL-6 was a biomarker of exacerbation-prone asthma. Subjects with high IL-6 also had a higher BMI and more frequent comorbid conditions, including hypertension and diabetes (Peters et al., 2016). Furthermore, Th1 polarization correlated with leptin, an adipokine associated with obesity-mediated inflammation, and IL-6, a cytokine downstream in the leptin inflammatory pathway, suggesting that obesity was driving the Th1 systemic inflammation in obese children with asthma (Rastogi et al., 2012). Although the number of classical and patrolling monocytes in obese children with asthma did not differ from that in obese children without asthma, the Th1/Th2 ratio inversely correlated with classical monocytes and directly correlated with patrolling monocytes uniquely in obese children with asthma and not in obese children without asthma, suggesting that obesity-mediated inflammation was more robust in obese children with asthma than in obese control subjects without asthma (Rastogi et al., 2015).

Macrophages act an important role in the pathogenesis of obesity-related asthma. In macrophages, the M1 type plays a pro inflammatory role (they can secrete a large number of pro-inflammatory cytokines, such as IL-1β, inducible nitric oxide synthase (iNOS), tumor necrosis factor-a (TNF-a)), and the M2 type has an anti-inflammatory effect (they mainly producing anti-inflammatory factors, such as IL-10, transforming growth factor-β (TGF-β), arginase 1 (Arg1)). Hypertrophic adipocytes and their secreted cytokines in obese patients lead to the conversion of M2-type to M1, this resulted in a shift from Th2-type helper cells to predominantly Th1(secretes IL-2, IFN-γ, TNF-α, et al.), Th17 (secretes IL-17A, IL-17F, IL-22, et al.), and CD8^+^T (secretes IFN-γ, IL-4, IL-22, et al.) cells. Inflammatory cytokines can reach the lung through the circulatory system and cause airway inflammation and airway hyperresponsiveness (Periyalil et al., 2018). Studies showed that airway neutropenia is a feature of obesity-related asthma, and lung neutrophil accumulation is associated with Th17 cells (Apostolopoulos et al., 2016). Obesity-related asthma patients have a large number of neutrophils and fewer eosinophils in their sputum, suggesting it may not be related to allergens. Studies found that macrophages can cause inflammation in obese mice, causing macrophages to secrete interleukin-1 (IL-1), induction of type 3 innate lymphocyte activation, and secretion of interleukin-17A (Interleukin-17A, IL-17A) to induce AHR and airway inflammation, which is the key mechanism of obesity-related asthma. ILCs play an important role in the inflammation of asthma. A mouse model showed that obese mice exposed to ozone had higher levels of IL13-producing ILC2 cells than lean mice (Mathews et al., 2017). Obesity-related asthmatic patients may lack the protective type 2 innate lymphoid cells and be replaced by type 3 innate lymphoid cells (Tashiro and Shore, 2019). A study found that obese individuals with asthma induced an increase adipose tissue inflammation compared with healthy controls, and metabolic dysfunction is more severe in obese patients. Leptin levels in obese adolescents were inversely related to forced expiratory volume in the first second (FEV1), forced vital capacity (FVC), and FEV1/FVC. And leptin expression in visceral adipose in adults correlates with airway responsiveness. Leptin and adiponectin also play a role in exercise-induced changes in lung function (Chen et al., 2020; Bantulà et al., 2021). A mouse study showed that leptin injection resulted in serum immunoglobulin E (IgE) and higher levels of airway AHR, but it has not been researched as a specific target in asthma (Zheng et al., 2018).

The relationship between airway responsiveness and leptin expression has been reported, and leptin plays an important role in obesity-related asthma (Sideleva et al., 2012). In mice without asthma, obesity itself was not found to exacerbate eosinophilic inflammation in lung tissue, or AHR (Jung et al., 2013). However, in children and adolescents with asthma, obesity is associated with elevated serum leptin and tumor necrosis factor-alpha levels (Grotta et al., 2013). Research reported that mice fed with HFD had significantly reduced Th2 cytokine production following the induction of allergic airway inflammation. However, these effects on respiratory tolerance and lung immune function did not reduce IgE production or lung inflammation. These results indicate that HFD is inclined to impair airway immune cell responses to allergens but does not change the development of respiratory tolerance (Pizzolla et al., 2016).

Adipocytes can secrete an important adipokine: adiponectin. It has been reported that obese patients have lower levels of adiponectin, which has anti-inflammatory effects. Human bronchial epithelial cells also express adiponectin, and adiponectin levels in visceral adipose tissue from obese asthmatic patients were significantly reduced during bariatric surgery, whereas adiponectin levels were higher in subcutaneous adipose tissue 12 months after surgery (Sideleva et al., 2012). Among obese asthma patients, there were no differences in resistin levels between men and women. In addition, studies have found that asthma is connected with a higher resistin/adiponectin ratio. Obese asthmatic men have higher resistin/adiponectin ratios. Therefore, resistin may be associated with the phenotype of obese asthma (Pizzolla et al., 2016).

**Figure 1:**
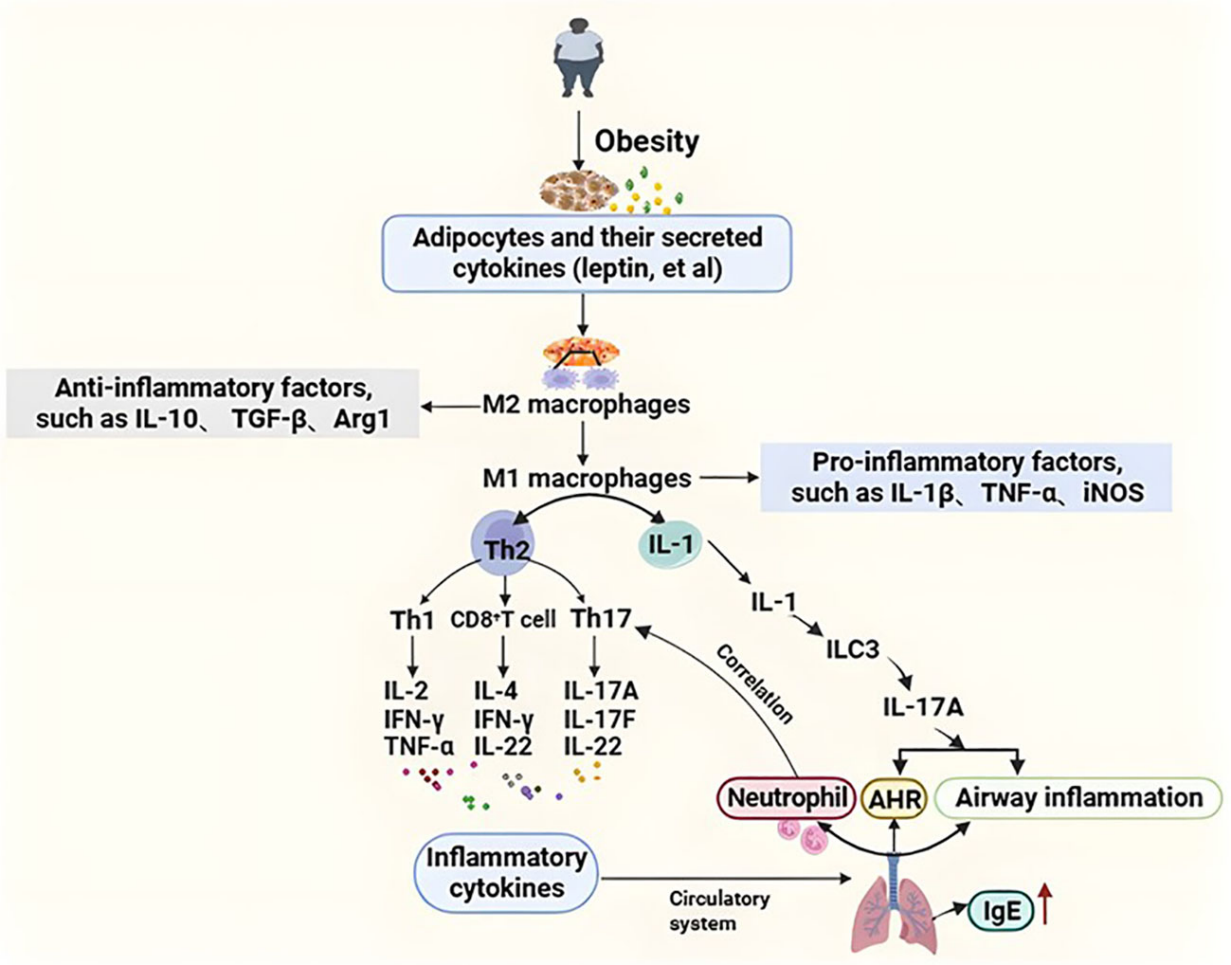
The inflammation mechanism of obesity-related asthma. Hypertrophic adipocytes and their secreted cytokines (e.g., leptin, etc.) in obese patients lead to the transformation of M2 (anti-inflammatory) type macrophages to M1 (pro-inflammatory) type, resulting in a shift from Th2-type helper cells to predominantly Th1, Th17, and CD8^+^ T cells. Inflammatory factors reach the lungs through the circulatory system causing airway inflammation and airway hyperresponsiveness. In addition, intrinsic lymphocytes are also involved in the pathogenesis of obesity asthma. Macrophages secrete IL-1, which induces the activation of type 3 intrinsic lymphoid cells to secrete IL-17A in order to induce AHR and airway inflammation, which is a key mechanism in the pathogenesis of obesity asthma. Airway neutrophilia is characteristic of obese asthma, and pulmonary neutrophil aggregation is associated with Th17 cells. Th1, T helper 1; Th2, T helper 2; AHR, Airway hyperresponsiveness; IgE, Immunoglobulin E; IL-1β, Interleukin-1beta; IL-2, Interleukin 2; IL-4, Interleukin 4;IL-10, Interleukin 10; IL-17A, Interleukin 17A; IL-17F, Interleukin 17F; IL-22 Interleukin 22; TGF-β, Transforming growth factor-beta; Tregs, Regulatory T cells; Arg1, Arginase-1; TNF-α, Tumor necrosis factor-α; IFN-γ, Interferon-γ; ILC3, Group 3 Innate Lymphoid Cells; iNOS, Inducible nitric oxide synthase.

As shown in **Figure 1**, low-grade systemic inflammation may be a mechanism in obesity and asthma, but it is unlikely to fully explain the association between obesity and asthma. More research is supposed to elucidate the underlying mechanism.

### 6.3 Mechanisms of metabolic disorders

Some studies suggest that metabolic syndrome is the real culprit behind asthma exacerbations. One study indicated that, compared with patients without metabolic syndrome, patients with metabolic syndrome had higher rates of poor asthma control after bariatric surgery. The complex interplay of obesity and systemic non-atopic inflammation can produce Th1 polarization, monocyte activation, and metabolic dysregulation through insulin resistance and dyslipidemia (Rastogi and Holguin, 2017). Furthermore, components of the metabolic syndrome were positively associated with impaired lung function (van Huisstede et al., 2013). Obese individuals and patients undergoing bariatric preoperative screening had higher proportions of monocytes and eosinophils compared with obese individuals without metabolic syndrome. Therefore, the presence of metabolic syndrome may affect lung function impairment by inducing systemic inflammation, especially mediated by blood eosinophils (van Huisstede et al., 2013). Recent studies have indicated that monocyte activation and Th1 polarization are associated with metabolic abnormalities in obese-asthmatic adolescents. Metabolic syndrome and insulin resistance have been linked to worsening lung function in overweight and obese adolescents and adults (Cardet et al., 2016; Tiotiu et al., 2018). And the study shows that metabolic syndrome could predict the incidence of asthma in adults after 11 years of follow-up (Brumpton et al., 2013). Therefore, if metabolic syndrome is aggressively treated, contemporary asthma may be treated more effectively.

Metabolic dysregulation plays a major role in many obesity complications, including asthma (van Huisstede et al., 2013). Hyperinsulinemia and hyperglycemia may lead to airway remodeling by airway smooth muscle proliferation and epithelial damage (Karampatakis et al., 2017). Pro-inflammatory cytokines associated with obesity, such as interleukin-6 (IL-6), play a critical role in the relationship between asthma severity, lung function, and metabolic syndrome. Recently, several novel methods, such as breath condensation analysis using nuclear magnetic resonance, have identified different metabolic signatures of obesity-related asthma, indicating that obesity-related asthma has a distinct pathogenic pathway compared to obesity or asthma alone (Bodini et al., 2017). Obesity increases oxidative stress; for example, one study found that obese adults with late-onset asthma had higher airway oxidative stress. And this is thought to be related to the reduced bioavailability of arginine, which is a substrate for the production of nitric oxide (NO, an endogenous bronchodilator). Thus, one of the causes of airway disease in obese patients may be reduced NO bioavailability (Giam et al., 2016; Grasemann and Holguin, 2021). Mouse models have indicated that lower L-arginine to asymmetric dimethyl arginine (ADMA) ratios can cause nitric oxide synthase uncoupling and elevated airway oxidative stress (Ahmad et al., 2010). Altered innate and adaptive immune responses, fat-related inflammation, and elevated oxidative stress may all lead to obesity-related asthma. A recent clinical study found that asthma treatment outcomes differed by poor metabolic health compared to metabolically healthy obesity, further supporting that the metabolic syndrome directly affects the obese asthma phenotype (Greiner and Hartwell, 2022).

The link between asthma and obesity was also associated at the genetic and epigenetic levels. In a twin study, 8 percent of the genetic component of obesity was associated with asthma, and phenotypic variation may be the result of genetic effects (Tang et al., 2014). In children and adults, gene polymorphisms located at the 1q31 locus and 17q21.2, respectively, were associated with a higher BMI in asthmatics (Hallstrand et al., 2005). Studies have found that the protein kinase C alpha (PRKCA) gene, leptin gene (LEP), and β3-adrenergic receptor gene (ADRB3) are associated with asthma and BMI (Ali et al., 2021). Children with different obesity and asthma statuses have different epigenome-wide deoxyribonucleic acid (DNA) methylation patterns (Rastogi et al., 2013). The beta-adrenergic receptor gene has been implicated in obesity-related asthma, and this gene has effects on both the respiratory and metabolic systems (Kuo et al., 2014). Studies found that a high-fat diet can induce the expression of the chitinase 3-like 1 gene (CHI3L1), and its expression product is associated with obesity and asthma.

### 6.4 Other mechanisms

NO has a dual effect on asthma. When NO is synthesized in an appropriate amount, it can relieve asthma symptoms by relaxing bronchial smooth muscle, but when it is synthesized excessively, its toxicity increases and asthma symptoms are aggravated. Previous studies indicated that low levels of serum vitamin D were associated with the worsening of severe asthma in children. Obesity seems to involve low circulating levels of vitamin D due to low sunlight exposure, low physical activity, and the intake of foods rich in vitamin D, volumetric dilution, and sequestration in adipose tissue. Since preadipocytes and adipocytes express receptors and are involved in the metabolism of vitamin D, it seems that low levels of this vitamin may be involved in adipogenesis and therefore in the development of obesity. This link is extremely important when considering diseases associated with obesity (Barrea et al., 2021). Prenatal vitamin D deficiency in pregnant women may be related to obesity in offspring, and maternal prenatal vitamin D supplementation reduces the risk of developing asthma in children within 3 years of age (Litonjua et al., 2016). The study also found that, compared with the non-asthmatic and non-obese control mice, the obesity-related asthma mice had the lowest vitamin D level, indicating that vitamin D may be related to allergy and inflammation, but its specific mechanism is unclear (Zhang et al., 2017). The hypothesis that vitamin D deficiency is associated with an obesity-related asthma phenotype is still somewhat controversial because vitamin D production is strongly influenced by the amount of air pollution present (which blocks ultraviolet radiation b that is necessary for vitamin D synthesis) (Hosseinpanah et al., 2010).

## 7 Relationship between obesity-related asthma and the microbiota

### 7.1 Obesity-related asthma and gut microbiota

The human digestive system contains trillions of microorganisms, and the gut microbiome is highly diverse. Alterations in gut microbiota composition and function can alter gut permeability, digestion, metabolism, and immune responses. The gut microbiota affects human physiology deeply and is critical for human gastrointestinal health, regulation of gastrointestinal development, metabolism and immune system, and defense against pathogens (Plaza-Díaz et al., 2015). Gut microbiota mostly acts as a biological barrier and is involved in immune regulation. Meanwhile, bacterial diversity can increase mucosal immune defense (Gray et al., 2017). But obesity may lead to gut microbiota dysbiosis and reduced bacterial diversity and result in an increase in *Firmicutes* and a decrease in *Bacteroidetes* (da Silva et al., 2013), which are major reasons for impaired gut barrier and immune function. In addition, dysbacteriosis can reduce the level of short-chain fatty acid (SCFA) in the body (Robles-Vera et al., 2020). SCFA is a classic histone deacetylase (HDAC) inhibitor to reduce inflammatory responses by inhibiting the NF-κB pathway (Koh et al., 2016), which can also repair lung epithelial cell damage caused by asthma. The gut microbiota may also affect the lungs by altering immune regulatory cells. As a result, this can lead to a range of problems, such as weight gain, insulin resistance, systemic inflammation, and asthma (Caesar et al., 2015). Studies found that an early reduction in gut microbial diversity can predict the development of asthma and lead to allergy through an imbalance of Th2/Th1 responses (Lugogo et al., 2018). Obesity-related inflammation and gut microbiota dysbiosis can lead to elevated intestinal mucosal permeability (Ahmad et al., 2017). LPS enters the blood circulation from the intestinal mucosa, resulting in endotoxemia. The underlying mechanisms involved are as follows: The binding of LPS to TLR4 may start the NF-κB pathway, resulting in the production of various cytokines, including TNF-α and IL-6 (Catrysse and van Loo, 2017). These cytokines act on the lungs and may contribute to the worsening of AHR and asthma. Obesity caused by a high-fat diet may increase asthma risk by altering gut bacteria. High-fat diet-induced microbial dysbiosis suppresses regulatory T cell function and increases Th2-induced airway inflammation through forkhead box P3 (Foxp3) promoters (Sharma and Cowan, 2021). Furthermore, obesity-induced gut dysbiosis leads to disturbances in cholesterol metabolism and decreases intestinal bile acid levels, thereby weakening the inhibitory effect of NLRP3. NLRP3 activation mainly induces IL-1β secretion through M1 macrophages, thereby inducing AHR, which is considered to be a major characteristic of asthma (Tolhurst et al., 2012). In addition, women who deliver by caesarean are often given prophylactic antibiotics, which can be detrimental to the baby’s gut microbiota and can exacerbate diseases such as obesity. Another study showed that ovalbumin-induced asthmatic mice had increased gut microbiota diversity when exposed to microbes early in life. These findings suggest that obesity due to a lack of microbial exposure early in life may contribute to the risk and exacerbation of asthma.

Diet can also influence the balance of the gut microbiota and be likely to improve obesity and asthma. Studies suggested that diet can affect inflammation and may produce clinically relevant endpoints in the form of increasing AHR, blocking IL-1β signaling with the IL-1 receptor antagonist anakinra, and reducing ILC3 and AHR in a mouse model of obesity (Kim et al., 2014). Research has reported that a high-fiber diet and propionic acid can prevent allergic lung inflammation caused by house dust mites in a manner dependent on the SCFA receptor Gpr41. Low-fiber-fed mice have reduced levels of circulating SCFAs and elevated allergic airway responses (Trompette et al., 2014). A high-fiber diet increased SCFA concentrations in the gut and circulation, thereby suppressing weight gain and allergic inflammation, which were dependent on gut microbiota metabolism (Martinez-Gonzalez et al., 2014). A study found that a high-fiber diet and SCFAs shaped the immune environment of the lung by regulating Treg differentiation and activation, and they also affected the severity of sexual allergy inflammation by inhibiting neutrophil production of pro-inflammatory reactive oxygen species (ROS) and TNF-α (Martinez-Gonzalez et al., 2014). Therefore, a diet rich in dietary fermentable fiber may have a positive effect on patients with obesity-related asthma. Among the functions of gut microbiota is fiber metabolization, it has been described that dietary fermentable fiber and SCFAs can outline the immunological lung environment and influence the severity of allergic inflammation (Trompette et al., 2014). Similarly, associations have been found between obesity (Dysregulation of microbiota in obesity leads to deficiency of SCFAs) and altered profiles of fecal SCFAs. Therefore, raising the concentration of short chain fatty acids (SCFAs), may be beneficial for patients with obesity-related asthma. Moreover, gastrointestinal bacteria can ferment what is not digested or absorbed to produce conjugated linoleic acid (CLA) such as trans-10 and cis-12 CLA. A study compared the weight-loss mechanisms of Caloric restriction (CR) and t10-c12 CLA, and found that the t10-c12 CLA reduced whole-body fat mass by decreasing all fat depots (visceral, inguinal, brown/interscapular), while CR lowered both whole-body fat and lean mass in obese mice. Nuclear Tbx-1, a marker of metabolically active beige adipocytes, was greater in the adipose of t10-c12 CLA-fed animals. Thus, weight loss achieved via t10-c12 CLA was primarily fat loss and increased numbers of cells with nuclear localization of the beige cell marker Tbx-1 (Yeganeh et al., 2017). A recent human trial indicated that CLA may work more effectively if used for prevention of body fat deposition and weight gain. To test this hypothesis, another study conducted 2 experiments using relatively old mice (older than 6 mo): experiment 1, supplementation of CLA during dietary restriction and experiment 2, supplementation during ad libitum feeding followed by restriction. The results showed in experiment 1, there were significant effects of diet restriction and CLA supplementation on body composition, while CLA decreased body fat content in ad libitum diet but not significantly during diet restriction. In experiment 2, CLA fed animals had body weights similar to restricted animals and CLA significantly reduced body fat (significantly lower than prior to and post restriction, or pair fed). This suggests that CLA exerted modulation of body fat independent of reduced food intake, CLA may be more effective at protecting against fat mass regain following weight loss than as a weight loss treatment (Park et al., 2007). *Bifidobacterium*, *Bifidobacterium pseudolongum*, and *Bifidobacterium breve* can convert free linoleic acid and α-linolenic acid to different CLA (Yeganeh et al., 2017). Consequently, CLA and CLA-related compounds represent a novel strategy for weight management that may improve the symptoms of obesity-related asthma.

It was found that asthma and obesity have an additive effect on microbiota changes in the pair, with significantly lower Shannon diversity in obese asthmatics (Michalovich et al., 2019). The ability of gut microbiota to influence interleukin-17A (IL-17A) may be particularly relevant to obesity-related asthma. Serum IL-17A in obese mice increased, and its mRNA expression in bronchoalveolar lavage fluid and lung tissue increased. IL-17A was also found to be elevated in obesity-related asthma patients (Marijsse et al., 2014). Furthermore, loss of congenital AHR was observed in obese mice deficient in IL-17A, and the gut microbiota may contribute to obesity-related asthma by altering IL-17A (Kim et al., 2014).

Metabolite produced by the gut microbiota of obese patients (such as endotoxin) may also contribute to asthma and affect the lungs. This metabolite can diffuse from the gut into the blood circulation, affecting the lungs and other target organs. Microbiota changes associated with circulating SCFA concentrations also influence allergic airway responses. High fat diet-fed mice had reduced circulating levels of SCFAs and elevated allergic airway responses (Trompette et al., 2014). Therefore, changes in gut microbial composition in obese patients may also influence metabolites in the lungs and airway function. Alterations of branched-chain amino acid metabolites have been observed in the blood metabolism of obese patients, and this small metabolite can diffuse in pulmonary capillaries and may affect lung function (She et al., 2013). Research found that the addition of probiotics to the diet reduced the expression of IL17A in the gut, liver, and adipose tissue induced by a high-fat diet (Moya-Pérez et al., 2015). At present, there are few studies related to obesity-related asthma and gut microbiota, so more follow-up studies are needed to explore and verify the specific relationship.

### 7.2 Obesity-related asthma and respiratory microbiome

Microbiota also present in the lungs of normal people. The airway microbiota may be altered in obesity-related asthma, and a recent bronchoscopy study of severe asthma patients found that BMI was related to changes in the composition of the airway microbiota and decreased eosinophils in lung tissue (Thuy et al., 2015). Whether these microbiome changes lead to eosinophilia or simply unrelated consequences of other changes seen in obesity is still unclear.

**Figure 2:**
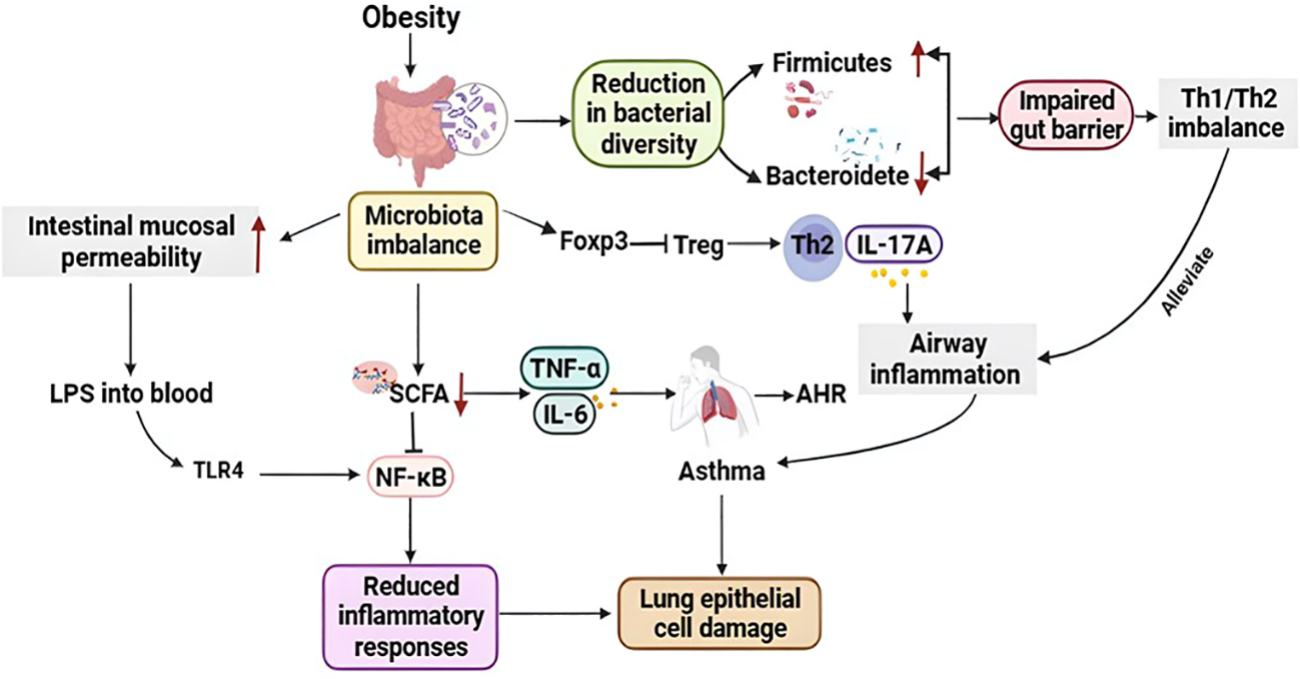
Relationship between obesity-related asthma and the microbiota. Obese individuals exhibit gut microbial dysbiosis, increase in Firmicutes and a decrease in Bacteroidetes, impaired gut barrier and immune function (Th1/Th2 imbalance); furthermore, gut microbial dysbiosis reduces short-chain fatty acid (SCFA) levels *in vivo*, which reduces inflammation by inhibiting the NF-κB pathway response, that also repairs asthma-induced lung epithelial cell damage. In addition, obesity-associated inflammation and dysregulation of the gut microbiota can lead to elevated intestinal mucosal permeability, and endotoxin from the intestinal mucosa into the circulation binds to TLR4 to initiate the nuclear factor-κB pathway, resulting in the production of multiple cytokines, including TNF-α and IL-6, which act on the lungs and may contribute to the exacerbation of AHR and asthma. In addition, microbial dysregulation inhibits Treg function via Foxp3 and increases Th2-induced respiratory inflammation. Th1, T helper 1; Th2, T helper 2; Tregs, Regulatory T cells; Foxp3, Forkhead box P3; IL-17A, Interleukin 17A; TNF-α, Tumor necrosis factor-α; IL-6, Interleukin 6; SCFA, Short chain fatty acid; LPS, Lipopolysaccharide; NF-κB, Nuclear factor kappa-B; TLR4, Toll-like receptor 4.

Ozone is a nonatopic asthma trigger, and obesity augments pulmonary responses to ozone. Depletion of the gut microbiota with mixed antibiotics might attenuate the response to ozone in obesity-related asthma. Besides, ozone-induced AHR was higher in germ-free mice than in wild-type mice, indicating a role for the microbiota (Tashiro et al., 2019). The study reported that childhood obesity and allergic asthma phenotype were significantly associated with the *Clostridium* unclassified family (Mbakwa et al., 2018). In a population of the koala, an uncultured *Clostridiales II* is inversely associated with BMI and body weight z-score (Gomez-Llorente et al., 2020). Studies have shown that bacterial colonization of the respiratory tract is associated with the development and severity of asthma. *Moraxella catarrhalis*, *Haemophilus influenzae*, or *Streptococcus pneumoniae* detected in the oropharynx of infants is associated with a significantly increased risk rate of recurrent wheezing and asthma in children (Hufnagl et al., 2020). A study found that H. pylori infection in the lungs of mice reduced their AHR, lung tissue inflammation, etc., and this protective effect disappeared after antibiotic treatment (Arnold et al., 2011). Patients with asthma with high airway microbial diversity also have higher AHR; specifically, the relative abundance of particular phylotypes, including members of the *Comamonadaceae*, *Sphingomonadaceae*, *Oxalobacteraceae*, and other bacterial families, was highly correlated with the degree of bronchial AHR (Huang et al., 2011). Sputum from patients with drug-resistant severe asthma contains relatively high levels of *Moraxella catarrhalis*, *Haemophilus influenzae*, and *Streptococcus pneumoniae*, which are associated with reduced lung function and elevated neutrophil and IL-8 concentrations in bronchoalveolar lavage (Campbell et al., 2022). The relationship between obesity-related asthma and the microbiota is shown in **Figure 2**.

## 8 Current status of obesity-related asthma treatment

### 8.1 Influence of obesity on the clinical treatment of asthma patients

The research discovered that the dosage of hormones, respiratory failure, hospitalization time, number of nebulizations, and other indicators of asthma treatment in children with obesity were significantly higher than those of normal-weight asthmatic children in the control group. Therefore, treatment of obese children with asthma needs to be highly valued and is more difficult than for normal-weight asthmatic children (Aragona et al., 2016). In a study of the impact of obesity on clinical treatment and lung function in asthmatic children, it was found that children with asthma who were clinically treated with inhalation therapy were better than obese children in terms of asthma control and pulmonary function indicators. The effective rate of asthma control was much higher than that of the obese group (Manuel and Luis, 2021). Epidemiological data show that obesity increases the incidence and severity of asthma. Obese patients have more difficult asthma control and are associated with higher rates of hospitalization and death. The incidence of shortness of breath and dyspnea in obese patients with asthma is 2.5 times that of non-obese patients with asthma. Growing research showed that, compared with non-obese asthma patients, asthma symptoms in obese patients are often difficult to control due to the ineffectiveness of standard treatment regimens and increased hospitalizations. In addition, the study found that adolescents with asthma and obesity were at higher risk of negative health and psychosocial difficulties compared to adolescents who were only overweight or obese (Fedele et al., 2014). Therefore, there is an urgent need to explore new therapeutic methods to solve the problem of obesity-related asthma.

## 9 Status of medication for obesity-related asthma

Most treatments are effective for asthma controllers (Lugogo et al., 2018). Studies showed that standard control therapy had little effect on obese adults, but rescue medications were effective (Razi et al., 2014). In adults, obesity-related asthma is less responsive to corticosteroids. A study showed that inhaled corticosteroids (ICS) or ICS combined with a long-acting beta2-agonist (LABA) were more difficult to achieve asthma control in obesity-related asthma than non-obese patients. In addition, increased small airway closure in obese patients may reduce the bioavailability of inhaled drugs (Lang et al., 2018a; Peters et al., 2018). Inhaled glucocorticoids remain the gold standard of therapy, despite lower efficacy in adults with obesity-related asthma (Lugogo et al., 2018). Compared with older children and adults, overweight or obese preschoolers have been found to have good control of their daily asthma symptoms with ICS therapy (Lang et al., 2018b). Cherry et al. found obese subjects with asthma used all asthma drug classes and higher doses of ICS compared to healthy-weight asthmatic subjects. But a better understanding of the factors driving increased drug use is needed to improve outcomes in this asthma subgroup (Thompson et al., 2021). ICS combined with LABA was superior to montelukast for lung function, asthma symptom scores, and rescue medication use (Di Genova et al., 2018). When theophylline was added to ICS, obesity-related asthma had a higher rate of asthma exacerbations than lean individuals (Lang et al., 2018b). The reason for the lower efficacy in obesity-related asthma is the overuse of asthma medications in more than 25 percent of patients (Di Genova et al., 2018). Some studies have shown obesity has no effect on response to omalizumab treatment; in other studies, obesity may reduce the effectiveness of omalizumab in terms of exacerbating asthma control scores, rescue therapy, and lung function (Sposato et al., 2018; Lentferink et al., 2019). Mepolizumab has an effect on the treatment of obesity-related asthma, especially in those with early-onset asthma (Ortega et al., 2014). There are no treatment response data for reslizumab, benralizumab, or dupilumab in obese eosinophilic asthma populations. Brodalumab, A new molecule, the anti-IL-17 receptor A monoclonal antibody can block IL-17A and IL-25 signaling, effective only in asthmatic patients with high IL-17, consistent with late-onset obesity-related asthma. There are also some promising targeted therapies probably providing an effective medical intervention strategy for the control of obesity-related asthma, such as microRNAs, TLR antagonists, and biologics of IL-1 and IL-6 (Ortega et al., 2014). Obesity-related asthma should be considered, including pharmacologic and nonpharmacologic treatments as well as approaches to identifying and treating comorbidities.

Older glucose-lowering drugs may lead to weight gain, while newer drug classes, sodium-glucose cotransporter 2 (SGLT 2) and glucagon-like peptide receptor agonists (GLP-1 RAs), target both weight loss and glycemic control. GLP1 agonists like liraglutide are a class of hypoglycemic agents with proliferator-mimetic activity approved for the treatment of type 2 DM. In subjects with DM, the use of GLP-1 RAs is associated with a significant reduction in glycated hemoglobin, accompanied by weight loss (Drucker, 2018). Subcutaneous liraglutide 3 mg once daily was found to be indicated for chronic weight management in adults with a BMI ≥ 30 kg/m2 or a BMI ≥ 27 kg/m2 and at least one weight-related comorbidity as an adjunct to a reduced energy diet and increased physical activity. In a double-blind, placebo-controlled, 20-week trial with an open-label orlistat comparator, 564 individuals were randomly assigned to 1 of 4 liraglutide doses (1.2 mg, 1.8 mg, 2.4 mg, or 3.0 mg) or placebo administered subcutaneously once daily or orlistat (120 mg) orally three times daily (Astrup et al., 2009). The mean weight loss was 4.8 kg, 5.5 kg, 6.3 kg, and 7.2 kg in the liraglutide 1.2 ~ 3.0 mg group, 2.8 kg in the placebo group, and 4.1 kg in the orlistat group. Weight loss was greater than 5% in the liraglutide 3.0 mg group (76%) compared with the placebo group (30%) or the orlistat group (44%) (Astrup et al., 2009). A 32-week double-blind randomized trial investigated whether liraglutide 3.0 mg reduced obstructive sleep apnea (OSA) severity compared to placebo in obese and non-diabetic patients. OSA severity measured by the apnea hypoventilation index (AHI) was found to be significantly lower in the liraglutide group compared to the placebo group, and liraglutide produced a greater mean percentage weight loss (5.7% vs.1.6%) (Blackman et al., 2016). Liraglutide achieved an average weight loss of 4-7 kg, with more than 50% of patients achieving a weight loss of 5% or more. These results led to regulatory approval of these drugs for weight loss in obese patients, with or without diabetes (Ortega et al., 2014). A recent case report reported the successful desensitization of two patients treated with GLP-1 receptor agonists (Yeğit et al., 2023). Based on the above effects of liraglutide, it may be useful for obese asthma patients and is a potential drug for the treatment of obesity-related asthma that needs further research and development.

SGLT-2 inhibitors are a new class of anti-hyperglycemic agents that have been approved for the treatment of type 2 diabetes mellitus (T2DM) (Yeğit et al., 2023). SGLT-2 inhibitors increase urinary glucose excretion by inhibiting renal glucose reabsorption, thus having anti-hyperglycemic and weight-reducing effects. The SGLT-2 inhibitor empagliflozin was recently reported to increase fat utilization and browning of white adipose tissue and to attenuate obesity-induced inflammation and insulin resistance by activating M2 macrophages (Xu and Ota, 2018). Both high and low doses of SGLT-2 inhibitors were associated with a reduced risk of 12 cardiopulmonary diseases (e.g., bradycardia, atrial fibrillation, hypertensive emergencies, asthma, chronic obstructive pulmonary disease, and sleep apnea syndrome) (Zou et al., 2022). Placebo-controlled cardiovascular (or cardiorenal) outcome trials reported that SGLT-2 inhibitors (OR, 0.59; 95% CI, 0.38-0.93) were significantly associated with a reduced risk of asthma compared with placebo, and SGLT-2 inhibitors may prevent asthma (Wang et al., 2022) and may also be useful in obese asthma due to their weight loss profile. In summary, perhaps SGLT-2 inhibitors could be approved for use in obese asthmatic patients without diabetes, and further confirmation using real data and mechanistic studies is needed.

## 10 Conclusions

Asthma and obesity are common chronic diseases that pose a serious threat to people’s quality of life, and obesity can exacerbate asthma. Obesity-related asthma is a specific asthma phenotype that is more severe, more symptomatic, poorly controlled, poorly responsive to treatment, and has a lower quality of life in obese patients compared to asthma alone. Obesity-related asthma is triggered by multiple mechanisms (e.g., low-grade chronic inflammation, metabolic syndrome, excess NO synthesis, and vitamin D deficiency), and it has a relationship with gut and airway microbes. Dysbiosis and reduced bacterial diversity in obesity-related asthma leads to impaired gut barrier and immune function. In addition, dysbiosis reduces SCFA levels, exacerbating inflammatory responses and AHR; the gut microbiota may also affect the lungs by altering Th17 cells; and CLA and CLA-related compounds produced by gastrointestinal bacteria metabolizing food may ameliorate obesity-related asthma symptoms.

In adults, obesity-related asthma responds poorly to corticosteroids. Although inhaled glucocorticoids are less effective in adults with obesity-related asthma, they remain the gold standard therapy; overweight/obese preschoolers have good control of daily asthma symptoms with ICS therapy compared with older children and adults; among treatments with monoclonal antibodies, omalizumab therapy is somewhat controversial; mepolizumab is effective in obesity-related asthma; the new molecule Brodalumab is effective only in asthmatics with high IL-17; liraglutide and SGLT-2 may be useful in obesity-related asthma and are potential drugs, but further research and development are needed. Currently, a variety of monoclonal antibodies have shown preliminary effects in the treatment of obesity-related asthma; however, there is still a need to find better drugs.

Most current research has focused on the negative effects of obesity on asthma. Research on the microbiota in obesity-related asthma is also very limited, so more and more in-depth animal and clinical obesity-related asthma models are needed in the future to elucidate its specific mechanism and guide the correct clinical medication. I hope that in the future there will be more in-depth research to explore the microbial mechanism of obesity-related asthma and new clinical drugs to reduce the suffering of obesity-related asthma patients, improving their quality of life.

## Author contributions

JH: Formal analysis, Investigation, Resources, Writing – original draft. XZ: Investigation, Writing – review & editing. BD: Investigation, Writing – review & editing. HT: Writing – review & editing, Formal analysis. QL: Writing – review & editing, Investigation. JZ: Writing – review & editing, Formal analysis, Supervision. HS: Supervision, Writing – review & editing, Visualization. XS: Supervision, Writing – review & editing.
